# Hartnup Disease Masked by Kwashiorkor

**DOI:** 10.3329/jhpn.v28i4.6049

**Published:** 2010-08

**Authors:** Zerrin Orbak, Vildan Ertekin, Ayse Selimoglu, Nebahat Yilmaz, Huseyin Tan, Murat Konak

**Affiliations:** ^1^ Department of Pediatric Endocrinology and Metabolism; ^2^ Department of Pediatric Gastroenterology and Nutrition; ^3^ Department of Pediatrics, Faculty of Medicine, Ataturk University, Erzurum, Turkey

**Keywords:** Acrodermatitis, Case studies, Hartnup disease, Kwashiorkor, Turkey

## Abstract

This report describes an 11-month old girl with Hartnup disease presenting with kwashiorkor and acrodermatitis enteropathica-like skin lesions but free of other clinical findings. This case with kwashiorkor had acrodermatitis enteropathica-like desquamative skin eruption. Since zinc level was in the normal range, investigation for a metabolic disorder was considered, and Hartnup disease was diagnosed.

## INTRODUCTION

Hartnup disease is an autosomal recessive hereditary disorder characterized by a defect in renal tubular re-absorption and intestinal transport of a group of mono-amine-monocarboxylic amino acids (neutral amino acids) (1,2). Although a biochemical disorder is always present, the clinical manifestations of Hartnup disease are intermittent and variable. Some asymptomatic cases have been recognized only through routine screening (3,4). In this article, we report a case with Hartnup disease presenting with kwashiorkor and acrodermatitis-like skin lesion.

### Case report

An 11-month old girl was admitted with severe kwashiorkor, acrodermatitis enteropathica, diarrhoea, and urinary tract infection. She had a three-week history of diarrhoea and four-day history of fever. She was born at term, following an uneventful pregnancy and delivery. She was almost exclusively breastfed during 11 months. Intake of solid foods was very poor. In the medical history, there was nothing eventful until three weeks before admission. Her parents were uneducated, appeared unaware of some basic elements of childcare, and their children did not receive regular medical care. They had not noticed when she began to show growth failure and insufficient weight gain. The parents were first cousins. They and their first child were healthy.

On examination, weight (6.470 kg), length (61 cm), and head circumference (42 cm) of the study girls were below the third centile for age. She had the typical features of kwashiorkor (generalized oedema, hypopigmented skin lesion on pretibial area, rotund sugar baby appearance, diaper dermatitis, abdominal distention, irritability, and thin, sparse hair) and rickets (obvious rachitic rosary, flaring of the wrists, craniotabes). Gross motor milestones were slightly retarded. There was no ataxia and nystagmus.

Laboratory evaluation was remarkable for a serum albumin of 2.6 g/dL, urea nitrogen of <6 mg/dL, and normocytic anaemia (7.5 g/dL). The serum alkaline phosphatase was markedly elevated, phosphorus was low, calcium was low normal, 25(OH)-vitamin D level was low, and the parathyroid hormone level was markedly elevated. Serum vitamin B_12_ and folate levels were normal. Evaluation for other causes of hypo-albuminaemia was negative.

Therapy for kwashiorkor was instituted, including gradual refeeding, initially via a nasogastric tube with a milk-based paediatric nutritional supplement. Supplements of vitamin D and calcium were provided. The oedema began to resolve after nutritional support. Serum zinc level was evaluated because an acrodermatitis enteropathica-like desquamative skin eruption persevered in the perineal areas (Fig.). Zinc level was in the normal range, and so, further investigation for a metabolic disorder was considered. After urine and blood samples were sent to the laboratory, our patient died on the seventh day of admission due to pneumonia. Chromatography of urinary amino acids revealed massive generalized neutral aminoaciduria and indicanuria. These amino acids were detected at low levels in the serum. Based on these findings, the diagnosis of Hartnup disease was made after her death.

**Fig.**
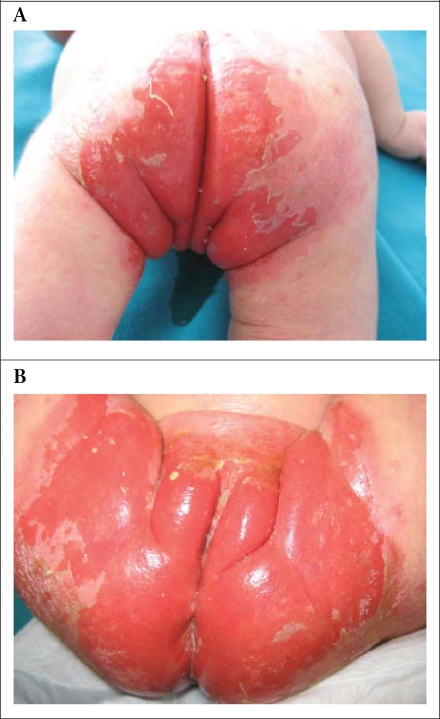
Acrodermatitis enteropathica-like skin lesion in the perianal region (A: Posterior view and B: Anterior view)

## DISCUSSION

Since the first description of the syndrome in several members of the Hartnup family in 1956 (5), an excessive number of patients who fulfil the biochemical diagnostic criteria have been reported. The classical clinical symptoms—pellagra-like dermatitis and neurological involvement—closely resemble those of nutritional niacin deficiency. They probably reflect deficient production of the essential tryptophan metabolites, particularly of nicotinamide. However, most patients are asymptomatic, possibly because the necessary amount of tryptophan is absorbed in oligopeptide form or because their niacin intake is sufficient (2).

Hartnup disease is a disorder characterized by a defect in renal tubular re-absorption and intestinal transport of a group of neutral amino acids. It leads to a specific aminoaciduria and to the retention of the same specific amino acids, including tryptophan in the intestine (1,2). Most affected individuals also excrete excessive amounts of indolic compound, which originate in the gut from bacterial degradation of unabsorbed tryptophan (1). The hallmark of kwashiorkor is a low serum albumin of dietary origin (6). The pathogenesis of kwashiorkor and peripheral oedema is likely multifactorial in our patient. The aminoaciduria and the defect of absorption of several amino acids were not compensated due to a limited protein intake, and as a result, kwashiorkor developed. Ozalp *et al*. described a five-year old girl with Hartnup disease presenting with hypo-albuminaemia and oedema without other clinical findings but did not report protein-energy malnutrition in their patient (7).

Reduced intestinal absorption and urinary loss of tryptophan lead to reduced availability of this amino acid for the synthesis of niacin. It is believed that the main classical clinical abnormalities are a consequence of niacin deficiency. These include a pellagra-like skin rash and reversible episodes of neurologic dysfunction (1). The most dramatic expression of the neurological involvement is cerebellar ataxia. Intention tremor, nystagmus, diplopia, persistent headache, and psychiatric symptoms may be present (8). Patients have been reported with developmental retardation but no other symptoms (9). Our patient had slightly delayed gross motor milestones. Also, it is known that the clinical manifestations are intermittent and variable, and they show a tendency towards spontaneous improvement with age (1).

A striking skin-rash is usually the first to become apparent (1,2). It occurs in the late infantile or juvenile period and occasionally in early infancy. The rash is identical to that seen in pellagra, dietary niacin deficiency. A red, scaly rash appears over the face, neck, hands, external surface of the arms, and dorsal surface of the feet. The skin is photosensitive, and the typical rash appears after exposure to the rays of sunlight (1). It is interesting that our patient did not show any clear signs of niacin deficiency despite hypo-albuminaemia. Skin lesions in our case were of the acrodermatitis enteropathica-type, and these were different from the typical skin eruptions of Hartnup disease. Acrodermatitis enteropathica lesions as a result of zinc deficiency are frequent in kwashiorkor. We think that acrodermatitis enteropathica-like lesions without hypozincaemia in protein-energy malnutrition should be a stimulus for investigating for Hartnup disease. Other than exposure to the sunlight, it is also known that fever, diarrhoea, inadequate diet, or psychological stress may precipitate the symptoms (2). Scriver *et al*. reported an eczematoid eruption over the body and thighs associated with oedema, followed by a prolonged episode of diarrhoea in one of 21 affected individuals identified through screening of newborns (10).

We know that all neutral amino acids are lost to a variable degree in urine and stool as a consequence of the membrane-transport abnormality. Although our patient had malnutrition and insufficient food intake, Hartnup disease has provoked a progression of kwashiorkor.

Consequently, Hartnup disease is manifested by a wide clinical spectrum. Most patients remain asymptomatic. The association of kwashiorkor and acrodermatitis enteropathica-like lesions, especially during infancy, may be accepted as one of the indications for systematic evaluation for some inborn errors of metabolic diseases in particular, for Hartnup disease. Malnutrition and a low-protein diet are the primary factors that contribute to morbidity (11). Based on our experience with this patient, we re-emphasize the importance of early diagnosis and effective treatment of the disease.

## References

[1] Lyon G, Adams RD, Kolodny EH (1996). Neurology of hereditary metabolic diseases of children, 2^nd^ ed..

[2] Simell O, Parto K, Näntö-Salonen K, Fernandes J, Saudubray JM, van den Berghe G (2000). Transport defects of amino acids at the cell membrane: cystinuria, lysinuric protein intolerance and hartnup disorder. Inborn metabolic diseases: diagnosis and treatment, 3rd rev ed..

[3] Seakins JWT, Ersser RS (1967). Effects of amino acid loads on a health infant with the biochemical features of Hartnup disease. Arch Dis Child.

[4] Wilcken B, Yu SJ, Brown DA (1977). Natural history of Hartnup disease. Arch Dis Child.

[5] Baron DN, Dent CE, Harris H, Hart EW, Jepson JB (1956). Hereditary pellagra-like skin rash with temporary cerebellar ataxia, constant renal amino-aciduria, and other bizarre biochemical features. Lancet.

[6] Carvalho NF, Kenney RD, Carrington PH, Hall DE (2001). Severe nutritional deficiencies in toddlers resulting from health food milk alternatives. Pediatrics.

[7] Ozalp I, Saatçi U, Hassa R (1977). A case of Hartnup disorder with hypoalbuminemia and edema. Turk J Pediatr.

[8] Nyhan WL, Barshop BA, Ozand PT (2005). Atlas of metabolic diseases, 2nd ed..

[9] Shih VE, Bixby EM, Alpers DH, Bartoscas CS, Their SO (1971). Studies of intestinal transport defect in Hartnup disease. Gastroenterology.

[10] Scriver CR, Mahon B, Levy HL, Clow CL, Reade TM, Kronick J (1987). The Hartnup phenotype: Mendelian transport disorder, multifactorial disease. Am J Hum Genet.

[11] Lidija Kandolf Sekulovic L, Karadaglic D, Stojanov L Hartnup disease.

